# Response to: A brief comment on the predictive value of myeloperoxidase-conjugated DNA level in patients with septic shock

**DOI:** 10.1186/s13054-018-2267-7

**Published:** 2018-12-18

**Authors:** Naoshi Takeyama

**Affiliations:** Department of Emergency and Critical Care Medicine, Aichi Medical University, Aichi, 480-1195 Japan

We read the letter from Li et al. with great interest. We appreciate their thoughtful comments and would like to address some of their concerns regarding our recently published article [1] in *Critical Care*.

Their first question was related to comorbidities and treatments. Thirty-two of our patients (58%) had at least one comorbidity, with frequent comorbidities being heart disease (28%), chronic obstructive pulmonary disease (14%), diabetes (13%), chronic kidney disease (8%), and cancer (4%). We agree that these comorbidities and treatments such as anticoagulants (discussed below) or fluid resuscitation may affect the plasma level of neutrophil extracellular traps (NETs) [2]. However, neutrophil activation and NET formation during sepsis would be much more prominent than in other inflammatory diseases, including atherosclerosis, deep vein thrombosis, ischemic heart disease, and collagen diseases. For example, the maximum plasma level of cell-free DNA (cf-DNA) was reported to be higher in ICU patients with infection than in other ICU patients [3].

The second issue they raised was the basis for selecting the assessment time points. Organ failure parameters obtained on day 1 and parameters of disseminated intravascular coagulation (DIC) obtained on day 3 were excluded from the “Results” section, but were presented in Additional files 1 and 2, respectively. On day 1, the plasma levels of MPO-DNA and cf-DNA were not correlated with parameters of coagulation or organ failure. Innate immunity, including phagocytosis and release of NETs, is the first line of defense against invading pathogens, but excessive NET formation leads to endothelial cell damage. Thus, an appropriate level of NET formation during the early stage of septic shock is required to reduce bacterial spread and prevent organ damage, but the “appropriate” plasma levels of MPO-DNA and cf-DNA remain unclear.

Finally, they raised the lack of a correlation between the DIC score and NET formation. Absence of a correlation between the plasma NET level and DIC score does not refute the immunothrombosis hypothesis proposed by Kambas [4] because anticoagulant therapy (antithrombin, recombinant thrombomodulin, protease inhibitors, etc.) is often performed for DIC in Japan. Such therapy would have influenced the findings of our study. Antithrombin administration was reported to reduce pulmonary NET formation due to lipopolysaccharide-induced endotoxemia [5], while serine protease inhibitors and recombinant thrombomodulin [6] also inhibit NET formation in vitro. In conclusion, we await further clinical studies exploring the role of NETs in the development of septic DIC.

## Additional files

Additional file 1: **Figure S2.** Correlations of MPO-DNA and cf-DNA levels with organ failure parameters. Correlations of MPO-DNA and cf-DNA levels with the MAP (A), the P/F ratio (B), and the SOFA score (C) on day 1 after the diagnosis of septic shock. (PPTX 90 kb)

Additional file 2: **Figure S3.** Correlations of MPO-DNA and cf-DNA levels with the platelet count and the DIC score. Correlations of MPO-DNA and cf-DNA levels with the platelet count (A) and the DIC score (B) on day 3 after the diagnosis of septic shock. (PPTX 74 kbb)

## Abbreviations

cf-DNA: Cell-free DNA
DIC: Disseminated intravascular coagulation
ICU: Intensive care unit
MPO-DNA: Myeloperoxidase-conjugated DNA
NETs: Neutrophil extracellular traps
