# Supplementary material for: Response to: A brief comment on the predictive value of myeloperoxidase-conjugated DNA level in patients with septic shock
Source: Crit Care. 2018 Dec 18;22:349. doi: 10.1186/s13054-018-2267-7 (PMC6299554; doi:10.1186/s13054-018-2267-7)
Supplement: Supplementary file 2 — Figure S3. Correlations of MPO-DNA and cf-DNA levels with the platelet count and the DIC score. Correlations of MPO-DNA and cf-DNA levels with the platelet count (A) and the DIC score (B) on day 3 after the diagnosis of septic shock. (PPTX 74 kbb) [file 13054_2018_2267_MOESM2_ESM.pptx]

## Slide 1
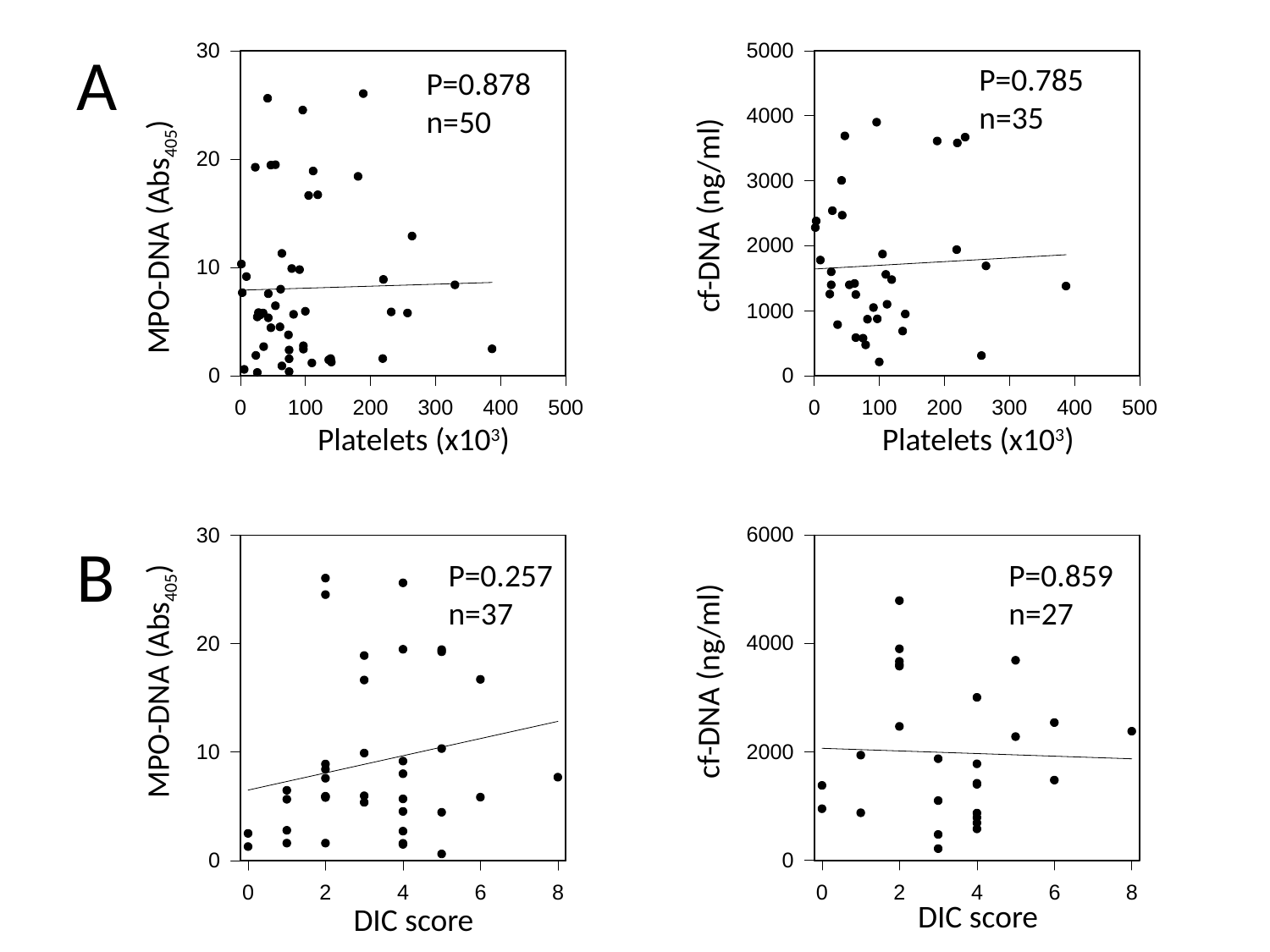

A
P=0.785
n=35
P=0.878
n=50
cf-DNA (ng/ml)
MPO-DNA (Abs405)
Platelets (x103)
Platelets (x103)
B
P=0.257
n=37
P=0.859
n=27
cf-DNA (ng/ml)
MPO-DNA (Abs405)
DIC score
DIC score
